# Environmental Presence of *Burkholderia pseudomallei* at Military Sites in an Endemic Region: Implications for Future Deployments and Studies

**DOI:** 10.4269/ajtmh.25-0542

**Published:** 2026-01-27

**Authors:** Kelly McCrory, Jacob G. Underwood, Sterling G. Perkins, Vanessa Rigas, Mark Mayo, Mirjam Kaestli, Ella M. Meumann, Andrew G. Letizia, Bart J. Currie

**Affiliations:** ^1^ Global and Tropical Health Division, Menzies School of Health Research, Charles Darwin University, Darwin, Australia;; ^2^ Marine Rotational Force Darwin, Darwin, Australia;; ^3^ Department of Infectious Diseases, Royal Darwin Hospital, Darwin, Australia;; ^4^ Microbiology Department, Territory Pathology, Darwin, Australia;; ^5^ Naval Medical Research Unit INDO PACIFIC, Sembawang, Singapore

## Abstract

The footprint of melioidosis is expanding globally, but its historical roots are in Southeast Asia and northern Australia. Melioidosis has long been described in military personnel deployed to melioidosis-endemic regions; however, the magnitude of the risk has not been quantified, and the nature of infecting events remains speculative. As with infections in the endemic population, the greatest concern is for inhalational melioidosis. Soil and air at four military locations in the highly melioidosis-prevalent environment of Darwin, Northern Territory, Australia, were sampled. *Burkholderia pseudomallei* (*B. pseudomallei*) was recovered from soil in all four sites but not from air samples. Genotyping revealed four *B. pseudomallei* sequence types (STs), with each ST recognized in human melioidosis cases from the region. Further systematic air sampling for *B. pseudomallei* is required both during the monsoonal wet season and under other circumstances, including aircraft with vertical takeoff and landing capabilities, to better understand the risk of inhalational melioidosis.

## INTRODUCTION

Melioidosis is increasingly being recognized globally, both through improved diagnostic capacity that unmask endemic regions and through the continued spread of the causative environmental bacterium, *Burkholderia pseudomallei* (*B. pseudomallei*), to new locations.^1^ Darwin is a coastal city in the tropical Top End of the Northern Territory of Australia with high incidence rates of melioidosis.^2^ Since 2012, a rotational force of US Marines has been deployed to Darwin during the dry season, which occurs from May through October, to support and work with the Australian Armed Forces on a collection of bases located within and around the city limits. In addition to exposure to snakes, crocodiles, jellyfish, blue-ringed octopus, and other environmental hazards, concerns have been raised about the potential risks of melioidosis during deployment.^3^ The basis for these concerns is historical links between melioidosis and military conflicts, as well as the potential for exposure to *B. pseudomallei* during military activities, with inhalational melioidosis being the greatest perceived threat.^4^^,^^5^ The aim for the present study was to undertake preliminary soil and air sampling from the environment in which the Marines work, to recover and characterize *B. pseudomallei*. A further aim was to place the environmental sampling findings in the context of previous studies from the Darwin environment, and to assess the current risk for Marines by quantifying cases of melioidosis in Australian military personnel in Darwin over the 35+ year Darwin Prospective Melioidosis Study (DPMS).^6^

## MATERIALS AND METHODS

During April and June 2024, soil and air samples were collected from four Australian military sites within and near metropolitan Darwin (A–D), where military personnel conduct air operations with vertical take-off and landing aircraft, form truck convoys to move personnel and equipment, and train at live firing ranges, all of which are activities that potentially expose them to environmental *B. pseudomallei*. Soil and air samples were processed using methods developed at Menzies School of Health Research, adapted from international consensus guidelines.^7^^–^^9^ Approximately 200 g of soil was collected from a depth of 10–30 cm using a sterile garden spade and placed into a sterile biohazard bag in a cooler bag to avoid sunlight exposure. In the laboratory, 20 g of soil was mixed with 20 mL of sterile water and placed in a shaking incubator (220 rpm) at 37°C for 2 days. A total of 10 mL of this mixture was then enriched in 30 mL of Ashdown’s broth containing colistin (50 mg/L) and incubated at 37°C under aerobic conditions for 7 days. Enriched broth was plated onto Ashdown’s agar with gentamicin (8 mg/L) on days 2 and 7 and incubated for 48 hours, and colonies resembling *B. pseudomallei* were further subcultured onto Ashdown’s agar for purity. For air samples, the Coriolis^®^ microbial air sampler (Bertin Technologies, Montigny-le-Bretonneux, France) was used, collecting each air sample run for 10–20 minutes into 15 mL Bertin collection liquid. The collection cone containing the liquid was then placed in a cooler bag to avoid exposure to sunlight. In the laboratory, the liquid was enriched in 30 mL of Ashdown’s broth and processed similarly to the soil samples.

Identification of *B. pseudomallei* was performed using type III secretion system real-time polymerase chain reaction (PCR) testing on Chelex DNA extractions of pure isolates on Ashdown’s agar.^10^ For whole-genome sequencing, DNA was extracted from all recovered *B. pseudomallei* and four bacterial isolates obtained via air sampling that were near-neighbor species to *B. pseudomallei* using the QIAGEN Qiamp DNeasy blood and tissue kit, with the addition of lysozyme (QIAGEN Pty LTD, Clayton, Australia). Multilocus sequence typing (MLST) sequence types (STs) for *B. pseudomallei* were derived in silico using the MLST assignment tool “mlst” (Seemann, T, https://github.com/tseemann/mlst) and the PubMLST website (https://pubmlst.org/). Near-neighbor taxa identification was performed using Kraken2 (v2.1.2).^11^

## RESULTS

A total of 52 soil samples were collected across the four military sites, with nine (17.3%) samples testing positive for *B. pseudomallei* (Table 1). ST36 was the most common ST recovered (*n* = 6), with three other single STs identified. None of the 47 air samples cultured *B. pseudomallei*, but air sampling revealed some near-neighbor bacteria. Four of these from three locations were identified as *Cupriavidus* spp.

**Table 1:** Sampling results from four Australian military sites within and near metropolitan Darwin

Site	Soil Sample Numbers	Number (%) of Soil Samples Positive for *B. pseudomallei*	Multilocus Sequence Types	Air Sample Numbers	Number of Air Samples Positive for *B. pseudomallei*
A	13	1 (8%)	562	11	0
B	20	3 (15%)	36, 36, 36	19	0
C	10	4 (40%)	36, 36, 36, 109	8	0
D	9	1 (11%)	552	9	0
Total	52	9 (17%)	–	47	0

*B. pseudomallei* = *Burkholderia pseudomallei*.

### Historical Darwin data.

The urban and surrounding rural environments of Darwin have undergone extensive soil and water sampling over the last three decades.^9^^,^^12^^,^^13^ The authors of one study were able to recover *B. pseudomallei* from 7 of 10 (70%) Darwin sports fields during the dry season.^14^ The top five STs of *B. pseudomallei* recovered from soils in the Darwin region include STs 36, 109, 132, 326, and 114. Sampling from military sites A, C, and D before construction of new accommodation units also revealed *B. pseudomallei*, including ST 36 (unpublished data). Air sampling has been more limited, with recovery of *B. pseudomallei* from air samples being only rarely successful.^8^

A study of environmental exposure in healthy participants during a muddy endurance challenge exercise in May (early dry season) revealed confirmed cutaneous melioidosis in 1/123 competitors.^15^ A serological study of 354 healthy residents of the Darwin Region, who self-identified as being active and having exposure to wet-season soils and surface water, revealed only 11 (3%) to be seropositive, with none developing confirmed melioidosis; however, several had self-limiting illnesses before the study that were considered potential resolving infections with *B. pseudomallei*, which cleared without antibiotics.^16^

Despite the frequent exposure of Darwin residents to *B. pseudomallei* during the dry season while participating in sporting and other recreational activities, it has been noted that confirmed melioidosis in healthy people is rarely attributed to such exposure.^6^^,^^14^ In addition, a review of the DPMS records revealed only six cases of melioidosis among Australian military personnel in Darwin over the initial 30-year period, representing 0.5% of 1,148 consecutive cases. Of these, all were previously healthy male patients, and five had cutaneous melioidosis following environmental exposure events during work activities, with each rapidly responding to therapy and making a full recovery. The final patient had neurological melioidosis attributed to oral or nasal inhalation from exposure during a wet-season storm. He had a protracted course and required prolonged therapy, but he made a good recovery and was able to return to work, although with a residual cranial nerve palsy.

## DISCUSSION

The results of the present study reveal that military personnel in Darwin may have frequent exposure to environmental *B. pseudomallei*, with the organism recovered from soil at each study location, despite the collections being performed during the dry season. Air sampling facilitated the recovery of other members of the *Burkholderiaceae*, but *B. pseudomallei* was not recovered. These findings are consistent with those of previous environmental surveys in the Darwin region, which have revealed that *B. pseudomallei* is widespread in local soils, including at some of the same sites as those in the current study.^9^^,^^12^^,^^13^ The environmental *B. pseudomallei* recovered in the present study, as well as from historical Darwin environmental sampling data from sites A, C, and D, include some of the most common STs observed in cases of melioidosis in Darwin,^6^ confirming the potential risk. Nevertheless, historically, few cases of melioidosis have been reported to date in Australian military personnel in Darwin, with 6/1,148 cases, suggesting that the risk is similar to that of the general population of Darwin, who have considerable occupational and recreational exposure to the local environment.^6^ The contribution of the current study lies in providing recent, site-specific environmental data that help characterize the persistence of *B. pseudomallei* at military facilities and the diversity of STs present, offering a baseline for future exposure assessments, especially during the wet season.

The recognition of melioidosis as a risk to military personnel deployed to melioidosis-endemic locations was noted among French troops in the Mekong River region.^4^ Subsequently, melioidosis was described in US troops in Vietnam, and the propensity for both chronic disease and activation from latency led to it being called “the time-bomb disease,”^5^ later characterized as the “Vietnamese time-bomb” in medical and lay literature from the United States.^17^ From serology studies, it was estimated that there were ∼225,000 servicepersons who returned to the US and may have been infected with *B. pseudomallei*.^18^ However, although latency up to 29 years has been documented,^19^ it has become clear that activation from latency is a rare event, accounting for less than 3% of all cases in the DPMS.^20^

Studies conducted across melioidosis-endemic regions have consistently revealed that most patients with melioidosis have predisposing clinical risk factors, with diabetes present in up to half of all cases.^1^^,^^6^^,^^21^ Although this recognition of melioidosis as an “opportunistic infection”^22^ is crucial for targeting prevention measures, it is also important to note that healthy people are still at risk, with 10–20% of melioidosis cases in northern Australia having no identified clinical risk factor.^6^^,^^21^

In addition to clinical risk factors, the route of infection has a major influence on presentation, severity, and outcome in melioidosis.^22^^–^^25^ Although both percutaneous infection and ingestion of *B. pseudomallei* can progress to systemic infection and severe sepsis, inhalational melioidosis is notable for resulting in rapidly progressive sepsis and a fatal outcome even in a healthy person. This finding is supported by animal inhalational melioidosis models^26^^,^^27^ and is analogous to the differential severity seen between cutaneous and inhalational infections in anthrax, plague, and tularemia.^28^

Inhalational melioidosis has been linked to exposure to wind and rain during severe weather events, as well as to specific occupational or recreational activities, such as the use of high-pressure hosing without face mask protection, lawn mowing, and the use of a “weed whacker” (“whipper snipper” or “string trimmer”) in the DPMS.^6^ A similar link to inhalational melioidosis was made for soldiers in Vietnam who were exposed to dust and uplifts in paddy fields generated by helicopter rotor blades,^5^ and helicopter-associated inhalation was implicated in melioidosis in a previously healthy tourist returned from Southeast Asia.^29^

A limitation of the present study is that sampling was only performed during the dry season, when the Marines were in Australia. In the DPMS, only 20% of cases of melioidosis occurred during the dry season, which spans 6 months (May through October),^6^ and risks for deployed military personnel will increase if the Marine rotational force expands its numbers and extends its presence in northern Australia to include the wet season. Advice for preventing percutaneous infection should focus on maintaining skin integrity and carefully cleaning and covering any skin lesions or wounds that breach it.

## CONCLUSION

What remains unclear is the risk of inhalational melioidosis posed by specific activities undertaken by military personnel in this highly endemic location. The authors of future studies should focus on linking *B. pseudomallei* environmental STs to clinical isolates from military personnel, conducting air sampling during military-specific activities (e.g., helicopter operations and munitions training), and quantifying aerosols generated during these events. The findings from such environmental sampling surveillance will help refine public health prevention advice for populations living in melioidosis-endemic regions and protect service members from infectious disease threats when they are deployed to these regions.
